# Histopathology and Molecular Genetics in Systemic Mastocytosis: Implications for Clinical Management

**DOI:** 10.3390/ijms23158772

**Published:** 2022-08-07

**Authors:** Francesca Crupi, Benedetta Sordi, Fiorenza Vanderwert, Francesca Gesullo, Andrea Amorosi, Francesco Mannelli, Raffaella Santi

**Affiliations:** 1 Centro Ricerca e Innovazione Malattie Mieloproliferative (CRIMM), AOU Careggi, 50134 Firenze, Italy; 2 Dipartimento di Scienze della Salute, Università Magna Grecia, 88100 Catanzaro, Italy; 3 Sezione di Anatomia Patologica, Dipartimento di Scienze della Salute, Università degli Studi di Firenze, 50121 Firenze, Italy

**Keywords:** systemic mastocytosis, bone marrow histology, misdiagnosis, mast cell disease criteria, *KIT*

## Abstract

The diagnosis of systemic mastocytosis (SM) is based on various clinical, dermatological, serological, and hematological findings but essentially relies on histological evidence of an abnormal increase in tissue-localized mast cells (MCs). The extra-cutaneous organ most frequently affected is the bone marrow (BM), and therefore, histological examination of trephine biopsy specimens of the iliac crest is mandatory on suspicion of SM. At microscopic examination, neoplastic MCs show aberrant morphology, usually with prominent spindling. Immunohistochemistry is a useful tool in the diagnosis of SM because mast cell (MC) infiltrates may be slight and scarce, in a mixed background of lymphohistiocytic cells, eosinophils, and plasma cells. Moreover, neoplastic MCs exhibit an aberrant phenotype. Recent evidence, largely derived from molecular genetics, has enhanced the diagnostic capability of SM, also providing the basis for adequate prognostic and therapeutic evaluation. The cases herein reported illustrate the variable clinical manifestations and disease course of SM, focusing on diagnostic and therapeutic challenges. In accordance with the World Health Organization (WHO) classification and the International Consensus Classification (ICC) systems, our findings emphasize the importance of an integrated diagnostic approach for SM, with proper application of diverse assessment methodologies in order to improve SM classification and treatment effectiveness.

## 1. Introduction

Systemic mastocytosis (SM) is a rare hematological neoplasm characterized by proliferation and accumulation of mast cells (MCs) in a variety of tissues, mainly skin and bone marrow (BM). Mastocytosis can be classified into cutaneous mastocytosis (CM), a skin-limited indolent disorder affecting mostly children, SM, an indolent to highly aggressive disease, occurring predominantly in adults, and the extremely rare mast cell sarcoma (MCS) [1,2]. In the World Health Organization (WHO) classification, SM is further clinically subdivided into bone marrow mastocytosis (BMM), indolent SM (ISM), smoldering SM (SSM), aggressive SM (ASM), SM with an associated hematological neoplasm (SM-AHN), and mast cell leukemia (MCL) [1]. According to the International Consensus Classification (ICC), BMM is recognized as an ISM variant and the SM-AHN subtype nomenclature has been changed to SM with an associated myeloid neoplasm (SM-AMN) [2]. The other SM subtypes are similarly defined in the WHO classification and in the ICC [1,2]. CM and ISM are by far the most frequent clinical variants; their course is indolent and life expectancy is practically normal, while ASM, SM-AHN/SM-AMN, and MCL are collectively identified as “advanced SM” and often manifest as aggressive, high-grade disease [3,4].

The main sign of SM is accumulation of MCs in BM, and biopsy of this tissue is therefore essential for the diagnosis. Histopathology plays a major role in the diagnosis of SM as reflected by the WHO and ICC diagnostic criteria, combining morphological, biochemical, cytofluorimetric, and molecular features [1,2]. In SM, MCs specifically present as compact multifocal aggregates of morphologically and immunophenotypically abnormal forms, and in most cases, with a diffuse, interstitial component [5]. Percentage and distribution of MCs throughout the BM are independent from the SM subtype. However, ample coalescing MC infiltrates have been found to be more common in the aggressive SM subtypes [6]. Unlike their normal counterparts, neoplastic MCs can vary in appearance, ranging from round to fusiform with long, polar cytoplasmic processes, occasionally exhibiting cytoplasmic hypogranularity, as well as atypical nuclei with monocytoid appearance [3,7,8]. Aberrant phenotype, e.g., CD25, CD30, and/or CD2 expression can also be detected at immunohistochemistry [2]. It should be emphasized that especially in non-advanced forms of SM, MCs may constitute an extremely low proportion of all nucleated BM cells at flow cytometry. In these cases, BM may show an increase in interstitial MCs, with no evidence of MC aggregates [9]. Consequently, it is mandatory that SM patients are referred to clinical specialists and expert pathologists in centers adequately equipped to offer the specific methodological approaches required [10,11].

The cases reported below are proposed as starting points for discussion on how various laboratory findings can be crucial for adequate clinical management.

## 2. Results

### 2.1. Case 1

**Figure 1:**
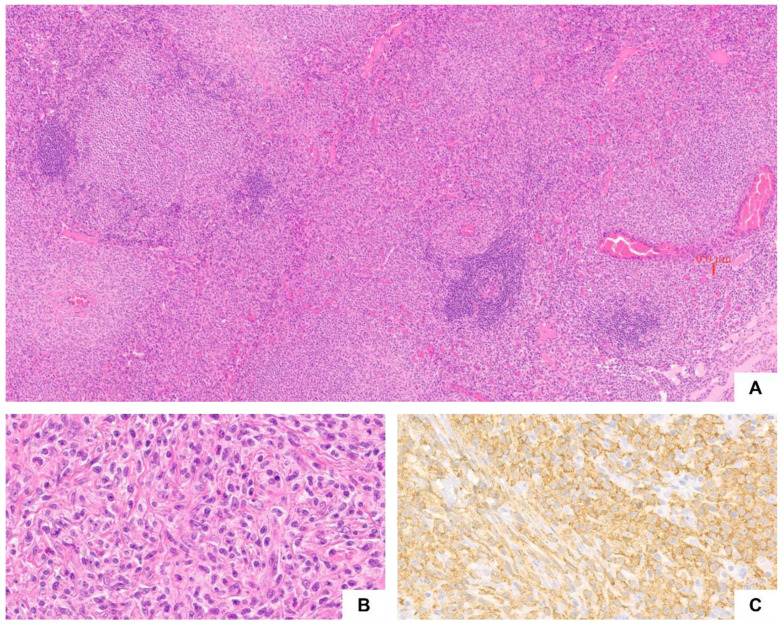
Case 1. Lymph node architecture was effaced by a diffuse, compact MC infiltrate, with few spared follicular structures. Slight collagen fibrosis, hyperplasia of small blood vessels and eosinophils were also seen ((**A**), hematoxylin an eosin, ×40). At higher magnification, MCs showed abundant pale cytoplasm and centrally located nuclei as well as spindle morphology ((**B**), hematoxylin and eosin, ×400). Immunohistochemically, MCs exhibited diffuse CD117 positivity ((**C**), ×400).

At SM onset, the patient was 60 years old and had a previous diagnosis of myelodysplastic syndrome with excess of blasts (MDS-IB1/MDS-EB) posed at the age of 48 years, for which he had undergone allogenic stem cell transplantation (HSCT) from an HLA-matched unrelated donor in 2007. Due to progressive loss of chimerism and occurrence of thrombocytopenia, the patient subsequently received four cycles of Azacytidine in 2013–2014, obtaining partial recovery of platelet count, around 75–85.000/mm^3^. In 2018, the patient experienced pancytopenia (WBC 3.9 × 10^9^/L; Hb 9.8 g/dL; Plt 12.000/mm^3^), accompanied by ascitic effusion, splenomegaly, and multiple abdominal lymph node enlargements detected at CT scan. On suspicion of malignancy, a mesenteric lymph node biopsy was performed, which documented a massive infiltration of MCs admixed with eosinophils, sparse follicles with benign germinal center, and minor fibrosis (Figure 1). BM examination revealed a multifocal MC infiltrate accounting for 30–40% of BM mononuclear cells, associated with dyserythropoiesis, dysmegakaryopoiesis with hypolobated megakaryocytes, and reticulin fibrosis grade 1 (MF-1). Moreover, *KIT* D816V mutation was detected (variant allele frequency, VAF: 8%) on mononuclear cells from peripheral blood (PB). Serum tryptase value was markedly increased (375 ng/mL). A diagnosis of ASM with associated hematological (myeloid) neoplasm was made. Next-generation sequencing (NGS) analysis documented the presence of additional somatic mutations of *MPL* (p.Y591X), *RUNX1* (p.R166X), *SRSF2* (p.P95R), and *TET2* (p.R1261G; p.E1319X; p.F1377C; p.H1386D). Midostaurin was started at the dosage of 100 mg BID based on a compassionate use protocol. The treatment was suspended due to exacerbation of thrombocytopenia (Plt 23.000/mm^3^) after four weeks of treatment and was restarted at the reduced dosage of 50 mg BID. Afterwards, neutropenia occurred and was effectively managed with weekly administration of granulocyte-colony stimulating factor (G-CSF). Restaging after three months of treatment with total body CT documented progression of disease, with worsening of ascites and lymphadenopathies. The patient died of pneumonia before alternative salvage treatment could be attempted.

### 2.2. Case 2

**Figure 2:**
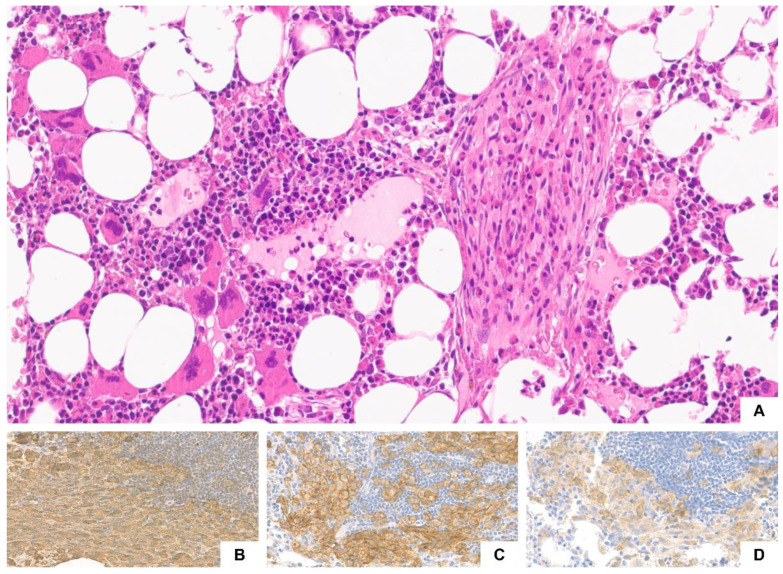
Case 2. A dense aggregate of spindle-shaped MCs admixed with eosinophils in the BM (on the right). Megakaryocytes were increased in number with several enlarged forms and loose clustering (on the left) ((**A**), hematoxylin and eosin, ×200). MCs positively staining for tryptase ((**B**), ×400), CD25 ((**C**), ×400), and CD30 ((**D**), ×400).

A 55-year-old woman presented with perimenopausal osteoporosis for which she began bisphosphonate therapy. In April 2021, after a few months of therapy, routine blood tests revealed an increased platelet count. Subsequent exams excluded reactive thrombocytosis, and the patient was thus referred to our center. In her medical history, there were no previous thrombotic events nor cardiovascular risk factors. At first evaluation, platelet count was mildly increased (between 600–700 × 10^9^/L). The patient underwent diagnostic workup with detection of *JAK2* V617F mutation by RT-PCR on PB (VAF: 9%). BM biopsy showed trilineage hemopoiesis with increased number/proliferation of large to giant megakaryocytes, often distributed in loose clusters, without any relevant increase in age-matched cellularity. The patient was diagnosed with essential thrombocythemia (ET) according to WHO/ICC criteria [1]. Moreover, as a concomitant finding, an increased number of MCs, mostly spindle-shaped and in multifocal dense aggregates, was highlighted (Figure 2). Immunohistochemistry revealed an aberrant MC phenotype, with expression of CD25. *KIT* D816V mutation was also documented (VAF: 0.1%) by droplet digital PCR (ddPCR) on BM. The diagnostic criteria for SM with an associated hematological (myeloid) neoplasm were fulfilled [1,2]. In view of the incidental diagnosis, we specifically explored the presence of mediator-related symptoms at the following follow-up without finding any of them, except for the previously reported osteoporosis. Based on the risk stratification of cardiovascular risk in ET patients according to IPSET-R [12], low dose aspirin (100 mg daily) was started. As regards SM, the patient was prescribed with epinephrine self-injector and entered regular follow-up.

### 2.3. Case 3

**Figure 3:**
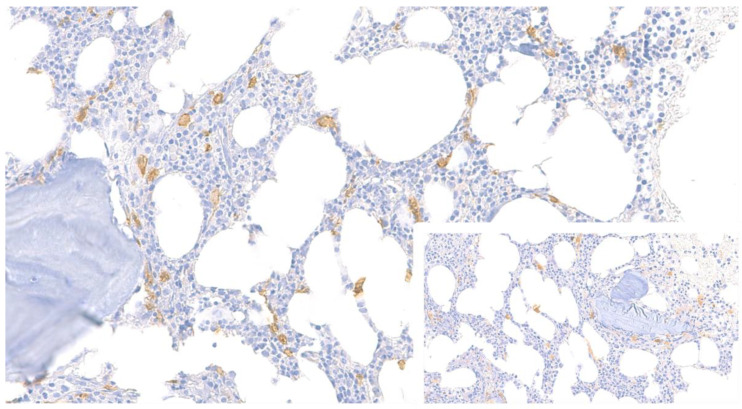
Case 3. Increased interstitial MCs highlighted by CD117 (×400) showing aberrant expression of CD25 (inset, ×400).

A 70-year-old man reported two episodes of anaphylaxis induced by hymenoptera stings. He did not present any other mediator-related symptom, neither skin lesions, organomegalies, nor lymph node enlargement. Following complete allergy work-up, hymenoptera venom immunotherapy was initiated and an epinephrine self-injectable disposal recommended. Blood tests showed a baseline serum tryptase value (BST) of 20.5 ng/mL; neither cytopenia nor liver alterations were observed. BM biopsy was then performed and histological examination showed MCs diffusely scattered in an otherwise normal BM, with frequent spindling and no cluster formation (Figure 3). *KIT* D816V mutation was detected both in PB (VAF: 0.05%) and in mononuclear BM cells (VAF: 0.03%). Multiparameter flow cytometry (MFC) analysis on BM samples documented aberrant expression of CD25 on MCs (representing 0.02% of the total cellularity). Cytogenetic examination showed loss of the Y chromosome in 3 of the 23 available metaphases. A diagnosis of BMM was then established [1,2]. A ddPCR based assay for *TPSAB1* copy number variation (CNV) highlighted a ββ;βαα genotype, thus confirming the presence of hereditary alpha tryptasemia (HαT). Dual X-ray absorptiometry (DXA) and endocrinological testing showed the presence of osteoporosis in the absence of bone fractures. Therapy with bisphosphonates was started.

### 2.4. Case 4

**Figure 4:**
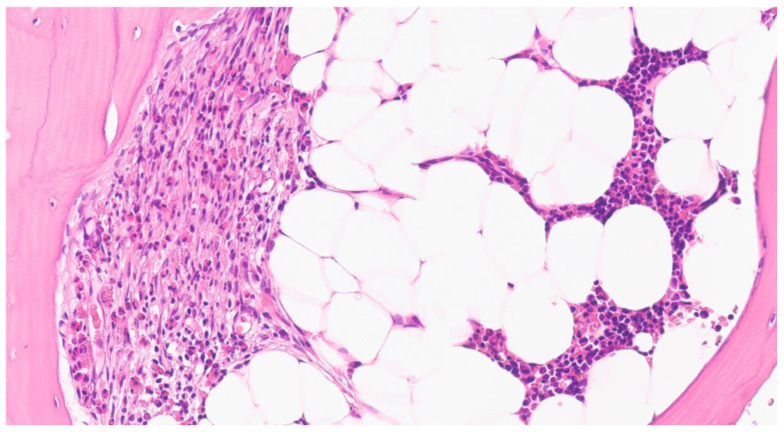
Case 4. Focal paratrabecular spindle-shaped MCs associated with numerous eosinophils in the BM (hematoxylin and eosin, ×200).

A 39-year-old woman was referred to our center in 2003 due to multiple episodes of loss of consciousness with no identifiable precipitating agent or event. She had a previous history of anaphylaxis due to hymenoptera sting (some months before admission) and complained of severe mediator-related symptoms, particularly of the gastrointestinal system. BST was 40 ng/mL. A first BM biopsy was performed in November 2009 and showed multifocal MCs infiltrates, accounting for 25% of cellularity. Flow cytometry revealed CD2 and CD25 expression on MCs, while *KIT* D816V mutation was negative using Sanger sequencing, RT-PCR, and ddPCR. She was initially treated with H1 and H2 antagonists and sodium cromolyn, obtaining only transient efficacy. After a few years, she experienced a significant decline in her quality of life due to recurrent hypotensive episodes accompanied by dizziness, palpitations, flushing, wheezing, abdominal pain, nausea, vomiting, diarrhea, fatigue, and short-term memory loss. Therefore, in November 2020, we repeated a disease assessment to rule out progression to an advanced variant (Figure 4). The results confirmed the indolent variant with absence of *KIT* D816V mutation by RT-PCR. Molecular evaluation was completed with sequencing of the entire coding gene (revealing wild-type *KIT*) and the NGS panel, which detected a variant of unknown significance in *ASXL1* gene (p.I593V, VAF: 10%). Although in the absence of C-findings, we decided to start with cytoreductive therapy due to the severe clinical manifestations and opted for Imatinib 100 mg per day. After 3 months of treatment, the patient achieved a significant improvement in symptom burden, also confirmed by a patient-reported outcome questionnaire developed specifically by REMA in the context of SM. The patient is now on continuous treatment with Imatinib maintaining optimal disease control.

## 3. Discussion

### 3.1. Case 1. Multi-Mutated ASM Treated with KIT Inhibitors

Regardless of category, all SM patients eventually increase their tissue MC burden. Considerable MC numbers mostly occur in patients with long-standing indolent mastocytosis or aggressive forms of the disease. Although increased in size, lymph nodes, liver, and spleen are rarely biopsied in routine management of mastocytosis unless significant organ dysfunction is evident or malignancy is suspected, as in the case herein reported [13]. Lymph node enlargement due to SM reveals dense, compact MC infiltrates, which often have a granulomatous appearance. Indeed, lymph nodes may contain abundant MCs, especially within the sinuses, in a variety of reactive and neoplastic disorders [14]. Although the paracortex is mostly affected by MC infiltrates in lymph nodes, any compartment can be involved. Eosinophilic infiltrates, fibrosis, and blood vessel proliferation may also be seen. Extensive lymphadenopathy in SM is rare but can pose a diagnostic challenge also for pathologists [15]. Paracortical distribution, clear cytoplasm, an associated vascular proliferation, and eosinophilia are all characteristics of MC infiltrates in lymph node tissues that mimic T-cell lymphomas, and once the lymphoid follicles are replaced by MCs, the configuration may well resemble follicular hyperplasia or lymphoma [16]. Mutations of genes usually associated with unfavorable prognosis in myeloid neoplasms are also harbored by a fraction of SM patients. Their presence provides meaningful insight into the course of the disease. Mutations in *SRSF2*, *ASXL1*, and *RUNX1* (summarized by the S/A/R acronym) have been shown to predict poor response to Midostaurin, a multikinase inhibitor being approved by regulatory agencies in 2017 for the treatment of advanced SM [17,18]. Furthermore, the different clinical manifestations (i.e., C-findings) seem to influence the probability of response to Midostaurin: in particular, portal hypertension and ascites generally portend minor and transient improvements, and so with lytic lesions [19]. These issues raise an important question about the role of *KIT* inhibition in multi-mutated, myeloid disorders, where this therapeutic modality can be expected to work on the SM component rather than on the AHN. This clearly supports the hypothesis of treatment combinations (i.e., *KIT* inhibitors plus conventional chemotherapy or hypomethylating agents) as an emerging prospect for this category of patients, to be verified within clinical trials.

### 3.2. Case 2. SM Associated with Myeloproliferative Neoplasm (MPN): Molecular and Clonal Heterogeneity

SM-AHN/SM-AMN covers a wide spectrum of clinical–biological conditions, and the estimation of its actual incidence is challenging: the reported frequency ranges from 5% to 40% of SM cases [20,21]. In general terms, SM-AHN/SM-AMN is likely underestimated especially in the absence of signs of aggressiveness. The associated neoplasm can range from acute leukemia to ET, with relative variability in disease course and prognosis [22,23]. A frequent association has been reported with chronic myelomonocytic leukemia (CMML), followed by myelodysplastic syndromes and myeloproliferative neoplasms. Lymphoid and plasma cell disorders are less frequently encountered and they do not share *KIT* mutation and/or other clonal genetic abnormalities with neoplastic mast cells [2,23]. On this basis, the ICC has excluded lymphoproliferative disorders from the definition of SM with an associated hematological neoplasm [2]. In the majority of patients (67%), the associated neoplasm is diagnosed concomitantly with SM, but the interval between the two diagnoses may vary from 3 months to 30 years [24]. Additionally, the frequency of *KIT* D816V mutation is dependent on the type of the associated neoplasm, being higher in CMML as compared to other neoplasms [25]. In the case we reported, SM was diagnosed concomitantly with a *JAK2* V617F-mutated MPN, an association that is generally featured by a particularly benign course [26]. Of note, in line with previous reports [27], the clinical picture of our patient did not include a differential diagnosis of mastocytosis. Conversely, patients with SM-MPN seldom present signs and symptoms of a specific MPN entity [21,22]. SM in BM can be obscured by the associated hematological neoplasm when only the hematoxylin and eosin-stained sections are examined [28]. Therefore, SM-AHN/SM-AMN can be considered a histological diagnosis dependent on the experience of the pathologists and their knowledge of MC proliferative disorders [27]. In general terms, it is increasingly recognized that SM-AHN/SM-AMN should be carefully characterized for both of its components and should not be inherently classified as an advanced variant simply as a function of association with an additional hematological disorder [3].

### 3.3. Case 3. A Carrier of HαT Diagnosed with SM

The clinical, histological, and molecular examination of our patient confirmed the presence of BMM, characterized by low burden of MCs confined to the BM, in the absence of skin lesions or visceral involvement [1,2]. Due to its peculiar features, BMM has probably been underestimated thus far, but more recent reports indicate that it may account for one-third [29] to one-fourth of cases diagnosed with ISM [30]. Our case recapitulates all main characteristics of BMM, from low BM MC infiltration to scarce mediator-related symptoms, except for anaphylaxis, often triggered by hymenoptera stings [31]. The disease course is generally benign, with high serum tryptase value (>125 ng/mL) and B-findings as the only independent risk factors for progression [30]. These results have prompted reconsideration of the definition of BMM, which requires the exclusion of the above variables, as well as absence of skin lesions and compliance with SM criteria [1,2,30,32]. Since patients with advanced SM may also lack skin lesions, clinical diagnosis of BMM can be challenging. In addition, as a consequence of an extremely low MC burden, serum tryptase levels may be only slightly higher or normal [31]. Hence, in BMM patients, symptoms suspicious of SM are heterogeneous, ranging from mediator-related symptoms to unexplained osteoporosis. Diagnosis of BMM is further complicated by the high percentage of BMM patients who do not meet the major histological criteria yet present with an interstitial BM pattern, as in our case. Notably, the spindle-shaped morphology criterion does not apply to MCs close to or lining blood vessels, endosteal surfaces, or nerve or fat cells [32]. Underestimation of MC number and degree of infiltration can be avoided with the use of immunohistochemical markers (Table 1).

Beyond the intrinsic characteristics of SM, recent findings have highlighted how the underlying genetic setting can predispose to the development of the disease and likely contribute to its clinical phenotype. Accordingly, in our case, we searched for and detected the presence of a CNV in the α-tryptase encoding sequence of *TPSAB1*, a genetic trait identified as HαT. This condition is characterized by an autosomal dominant inheritance, often associated with MC activation, elevated BST [33], and a higher-than-expected incidence in patients with SM, thus suggesting a role in facilitating disease development [34,35,36]. From a clinical standpoint, the presence of HαT has been associated with high risk of anaphylaxis, regardless of the underlying MC disorder [37,38,39]. As such, HαT is emerging as a robust biomarker for identifying patients at particularly high risk of life-threatening complications, a role that assumes further importance when considering the otherwise indolent course of the disease. Currently, the detection of *TPSAB1* CNV is not widely available and presents some technical issues due to the high degree of structural homology in the tryptase locus that preclude the direct sequencing by Sanger and Southern blotting. ddPCR is the assay of choice to overcome these limitations and provides absolute copy number detection of alpha and beta-tryptase sequences [37]. It is conceivable that the identification of HαT genotype could soon be integrated into the diagnostic evaluation of SM patients, in consideration of the synergistic effect on the triggering of severe reactions exerted by concomitant HαT and SM, a mechanism that deserves to be fully elucidated and possibly tackled. BST level > 20 ng/mL, a minor criterion of SM, should be adjusted in case of HαT [1,32].

### 3.4. Case 4. KIT D816V-Negative SM with Severe Clinical Symptoms

The *KIT* D816V mutation, located in the phosphotransferase domain (PTD) of the receptor, was detected in over 80% of SM patients, depending on disease subtype (ISM more frequently than ASM) and cell source (BM more sensitive than PB) [40]. Strongly associated with disease subtype and prognosis, *KIT* VAF does not correlate with the degree of BM infiltration by MCs [41]. Interestingly, non-MC-lineage cells in the BM were found to harbor differing levels of *KIT* mutation, which could explain this lack of correlation between VAF and the degree of MC infiltration [40]. Although the low sensitivity of Sanger (∼10%–20%) and NGS (∼5%) can generate false negative results, high-sensitive RT-PCR assays (∼0.04%) allow for the identification of *KIT* D816V in almost all mutated patients [42]. Besides *KIT* D816V, critical mutations causing ligand-independent activation of KIT and aberrant MC proliferation have been detected in SM and are considered a minor criterion in the actual classifications [1,2,32]. It is currently not standard clinical practice to screen for *KIT* mutations other than those involving D816; however, in the D816V-negative fraction, genotypic data can be useful for clinical management. Several non-D816V *KIT* mutations have been described which are generally divided into “regulatory” and “enzymatic pocket” types [43]. Among the latter, D816V confers chemoresistance to some TKIs, such as Imatinib and Dasatinib, while mutations in the regulatory domain, such as K509I or F522C, can determine exquisite sensitivity [44]. The literature data reported responses up to 40% in an SM patient with *KIT* WT status [45], thus making a therapeutic attempt rational in this setting, which may prove effective in controlling the disease, as in the patient we described.

## 4. Materials and Methods

### 4.1. Histology

BM trephine biopsies were fixed and decalcified using standard methods. Briefly, they were fixed for a minimum of 24 h in 10% neutral formalin and mildly decalcified for 6 h in edetic acid. Formalin-fixed paraffin embedded (FFPE) 2 μm tissue sections were prepared for conventional (i.e., hematoxylin and eosin, Gomori’s silver impregnation, and Perls’ Prussian blue reaction) and immunohistochemical stains.

Lymph node specimen was fixed in 10% buffered formalin and embedded in paraffin. Sections 3 μm in thickness were cut for routine histopathological examination and immunohistochemical studies.

### 4.2. Immunohistochemistry

Immunohistochemistry was performed with automated immunostainer (Ventana Discovery ULTRA, Ventana Medical Systems, Tucson, AZ, USA). Sections were deparaffinized in EZ prep (Ventana Medical Systems). Antigen retrieval was achieved by incubation with cell conditioning solution 1 (CC1, Ventana Medical Systems). Sections were then incubated with the following primary antibodies: anti-CD2 (Ventana Medical Systems), anti-CD3 (Ventana Medical Systems), anti-CD15 (Ventana Medical Systems), anti-CD20 (Ventana Medical Systems), anti-CD25 (Cell Marque, Rocklin, CA, USA), anti-CD30 (Ventana Medical Systems), anti-CD34 (Ventana Medical Systems), anti-CD68PGM1 (Diagnostic Biosystems, Pleasanton, CA, USA), anti-CD117 (Cell Marque), anti-Factor VIII-R Ag. (Cell Marque), anti-Glycophorin A (Cell Marque), anti-Ki-67 (Ventana Medical Systems), anti-Myeloperoxidase (Cell Marque), and anti-Tryptase (Cell Marque). Signal was developed using UltraMap diaminobenzidine antimouse or antirabbit detection kit (Ventana Medical Systems).

### 4.3. Molecular Biology

The presence of the *KIT* D816V mutation was analyzed using ddPCR assay [46]. The following types of samples were included in the study: whole PB or granulocytes isolated using Ficoll-Paque density gradient centrifugation and whole BM. A cell line HMC 1.2 heterozygous for the mutation D816V was used as a positive control. Negative controls included non-mast cell leukemia cell lines (NB4; HEL-60). The diagnosis of HαT was carried out by performing a ddPCR assay.

#### 4.3.1. DNA Extraction

For both analyses, DNA extraction was performed on Maxwell^®^ RSC Instruments using RSC Blood DNA Kit. DNA was extracted from 300 μL of whole PB in a volume of 50 μL of elution buffer.

#### 4.3.2. NGS

Extracted DNA was quantified using Nanodrop-2000 spectrophotometer (Thermo Fisher Scientific, Waltham, MA, USA) and Qubit dsDNA assay kit with Qubit fluorometer 2.0 (Thermo Fisher Scientific) to assess quality and suitability for library preparation. Libraries were prepared by the Ion Chef Instrument (Thermo Fisher Scientific), with an optimal amount of DNA of 50 ng, following the Ion Torrent Oncomine Research Assay user guide. Sequencing analysis was carried out using Ion 520 Chip (Thermo Fisher Scientific) and Ion Torrent S5 (Thermo Fisher Scientific) instrument. The obtained data were processed with the Ion-Reporter Software v.5.14 (Thermo Fisher Scientific). The Oncomine Myeloid Panel includes 17 full genes (*ASXL1*, *BCOR*, *CALR*, *CEBPA*, *ETV6*, *EZH2*, *IKZF1*, *NF1*, *PHF6*, *PRPF8*, *RB1*, *RUNX1*, *SH2B3*, *STAG2*, *TET2*, *TP53*, and *ZRSR2*) and 23 hotspot genes (*ABL1*, *BRAF*, *CBL*, *CSF3R*, *DNMT3A*, *FLT3*, *GATA2*, *HRAS*, *IDH1*, *IDH2*, *JAK2*, *KIT*, *KRAS*, *MPL*, *MYD88*, *NPM1*, *NRAS*, *PTPN11*, *SETBP1*, *SF3B1*, *SRSF2*, *U2AF1*, and *WT1*) involved in myeloid malignancies.

#### 4.3.3. Droplet Digital PCR

ddPCR was performed by Bio-Rad QX200 Droplet Digital PCR (Bio-Rad) and the data were analyzed using QuantaSoft software (Bio-Rad, Hercules, CA, USA). The presence of HαT was analyzed on genomic DNA with and without a restriction enzyme, using the PrimePCR ddPCR copy number reference AP3B1 (Bio-Rad). Handling DNA digestion strategies is important to understand and evaluate monoallelic copy number changes. The assay was designed with custom primers and probes for α- and β-tryptase obtained from published sequences [30]. The primers allowed amplification of both our regions, while each of the two probes was specific for one isoform: FAM for α and HEX for β-tryptase. For the *KIT* mutation in each ddPCR reaction, a FAM-labeled probe for the D816V mutation and a HEX-labeled probe for the *KIT* wild-type allele were used (Bio-Rad, UniqueAssayID dHsaMDV2010023). The total number of droplets detected in each ddPCR analysis exceeded 10,000 as requested. The final results are both expressed as copies/μL. The D816V mutation burden was calculated by dividing mutation droplets by (mutation droplets + wild droplets). All experiments were performed in duplicate wells.

## 5. Conclusions

Through a case-based approach, we aimed to illustrate clinical and pathological features of SM, as well as diagnostic and therapeutic challenges. Extra-cutaneous, extra-medullary SM localization encompasses several entities in the differential diagnosis, and immunohistochemistry is of help to confirm mast cell lineage and aberrant phenotype (Case 1). Despite the peculiar morphological and phenotypical anomalies of neoplastic MCs, pathological diagnosis of SM on BM biopsy may be challenging in the presence of another hematological neoplasm (Case 2) or when tissue tumor burden is low, as in BMM (Case 3). Although the *KIT* D816V mutation is a typical finding in SM patients, affecting prognosis and targeted drug therapy, its absence does not exclude the diagnosis and it is not linked to tumor tissue burden (Case 4). Besides describing different histomorphological aspects of SM, these cases also provide evidence of the broad range of clinical, biochemical, and molecular genetic features of this multifaceted disease. Integrated diagnostic approaches, involving the expertise of multiple disciplines and highly-specialized laboratory tests, are required for appropriate clinical management and treatment of SM patients.

## Figures and Tables

**Table 1:** Immunohistochemical markers employed in the diagnosis of SM. Of note, CD2, CD25, and CD30 expression in MCs can be detected by either flow cytometry or by immunohistochemistry.

Antibody	Pattern of Staining	Sensitivity for Mast Cells *	Specificity for Mast Cells *	Specificity for Neoplastic Mast Cells *	Comment
CD117 (*KIT*)	Membrane	+++	+	-	Proves MC lineage irrespective of normal/reactive or neoplastic status. Highly sensitive but not entirely specific for MCs since melanocytes, interstitial cells of Cajal, and hematopoietic stem cells also express CD117.
Tryptase	Cytoplasm	+++	++	-	Proves MC lineage irrespective of normal/reactive or neoplastic status. Highly specific for MCs.
CD2	Membrane	-	-	+++	Aberrantly expressed in neoplastic MCs, but its use in routine practice is limited due to variability among different cases and even within individual cases. CD2 is not specific for MCs and also stains T lymphocytes and a subset of NK cells.
CD25	Membrane	-	-	+++	Aberrantly expressed by neoplastic MCs. CD25 is not specific for MCs and also stains a subset of T lymphocytes.
CD30	Membrane	-	-	+++	CD30 is detectable in activated/proliferating lymphoid cells and in a distinct subset of tumors, including embryonal carcinoma, Hodgkin lymphoma, anaplastic large cell lymphoma, and extramedullary myeloid sarcoma. CD30 has been accepted as a minor diagnostic criteria in the WHO classification and in the ICC. CD30 is of help in the diagnosis of well-differentiated SM, usually negative for CD2 and CD25. CD30 does not qualify as a reliable grading marker in SM.

* +++ high; ++ moderate; + low; - none.

## Data Availability

Not applicable.
